# Strategic Decoy Peptides Interfere with MSI1/AGO2 Interaction to Elicit Tumor Suppression Effects

**DOI:** 10.3390/cancers14030505

**Published:** 2022-01-20

**Authors:** Yi-Ping Yang, Andy Chi-Lung Lee, Liang-Ting Lin, Yi-Wei Chen, Pin-I Huang, Hsin-I Ma, Yi-Chen Chen, Wen-Liang Lo, Yuan-Tzu Lan, Wen-Liang Fang, Chien-Ying Wang, Yung-Yang Liu, Po-Kuei Hsu, Wen-Chang Lin, Chung-Pin Li, Ming-Teh Chen, Chian-Shiu Chien, Mong-Lien Wang

**Affiliations:** 1 Department of Medical Research, Taipei Veterans General Hospital, Taipei 112, Taiwan; molly0103@gmail.com (Y.-P.Y.); leechilung101@gmail.com (A.C.-L.L.); cj093138@gmail.com (Y.-C.C.); 2 School of Medicine, College of Medicine, National Yang Ming Chiao Tung University, Taipei 112, Taiwan; chenyw@vghtpe.gov.tw (Y.-W.C.); pihuang@vghtpe.gov.tw (P.-I.H.); wllo@vghtpe.gov.tw (W.-L.L.); ytlan@vghtpe.gov.tw (Y.-T.L.); wlfang@vghtpe.gov.tw (W.-L.F.); wangcy@vghtpe.gov.tw (C.-Y.W.); yyliu@vghtpe.gov.tw (Y.-Y.L.); hsupokuei@yahoo.com.tw (P.-K.H.); cpli@vghtpe.gov.tw (C.-P.L.); mtchen@vghtpe.gov.tw (M.-T.C.); 3 Institute of Food Safety and Health Risk Assessment, College of Pharmaceutical Sciences, National Yang Ming Chiao Tung University, Taipei 112, Taiwan; 4 Institute of Pharmacology, National Yang Ming Chiao Tung University, Taipei 112, Taiwan; 5 Department of Health Technology and Informatics, The Hong Kong Polytechnic University, Hong Kong, China; aceliang@gmail.com; 6 Department of Neurosurgery, Taipei Veterans General Hospital, Taipei 112, Taiwan; 7 Department of Oncology, Taipei Veterans General Hospital, Taipei 112, Taiwan; 8 Department of Neurological Surgery, Tri-Service General Hospital and National Defense Medical Center, Taipei 114, Taiwan; uf004693@mail2000.com.tw; 9 Division of Oral and Maxillofacial Surgery, Department of Stomatology, Taipei Veterans General Hospital, Taipei 112, Taiwan; 10 Division of Colon & Rectal Surgery, Department of Surgery, Taipei Veterans General Hospital, Taipei 112, Taiwan; 11 Department of Surgery, Taipei Veterans General Hospital, Taipei 112, Taiwan; 12 Division of Trauma, Department of Emergency Medicine, Taipei Veterans General Hospital, Taipei 112, Taiwan; 13 Department of Critical Care Medicine, Taipei Veterans General Hospital, Taipei 112, Taiwan; 14 Department of Physical Education and Health, University of Taipei, Taipei 111, Taiwan; 15 Chest Department, Taipei Veterans General Hospital, Taipei 11217, Taiwan; 16 Institute of Biomedical Sciences, Academia Sinica, Taipei 115, Taiwan; wenlin@ibms.sinica.edu.tw; 17 Department of Medical Education, Taipei Veterans General Hospital, Taipei 112, Taiwan; 18 Division of Gastroenterology and Hepatology, Department of Medicine, Taipei Veterans General Hospital, Taipei 112, Taiwan

**Keywords:** decoy peptide, MSI1 C-terminus, MSI1/AGO2 disruption, protein–protein interaction, tumor suppression

## Abstract

**Simple Summary:**

Peptide drugs that can specifically target undesirable protein–protein interactions that lead to oncogenic developments have emerged as the next era of future medicine for cancers. To combat GBM tumor progression, our study offers an alternative therapeutic strategy via targeting the protein–protein interaction between MSI1 and AGO2 with synthetic peptides identified from the C-terminus of MSI1 in peptide arrays. Our present data revealed for the first time that peptidic disruption to the MSI1/AGO2 complex known for promoting cancer stemness and progression could lead to encouraging therapeutic efficacy at both in vitro and in vivo levels. The significantly suppressed tumor growth and prolonged survival rates in PDX tumor models by decoy peptides evidently provided a new rationale for stratifying patients with MSI1/AGO2-targeted therapeutics.

**Abstract:**

Peptide drugs that target protein–protein interactions have attracted mounting research efforts towards clinical developments over the past decades. Increasing reports have indicated that expression of Musashi 1 (MSI1) is tightly correlated to high grade of cancers as well as enrichment of cancer stem cells. Treatment failure in malignant tumors glioblastoma multiform (GBM) had also been correlated to CSC-regulating properties of MSI1. It is thus imperative to develop new therapeutics that could effectively improve current regimens used in clinics. MSI1 and AGO2 are two emerging oncogenic molecules that both contribute to GBM tumorigenesis through mRNA regulation of targets involved in apoptosis and cell cycle. In this study, we designed peptide arrays covering the C-terminus of MSI1 and identified two peptides (Pep#11 and Pep#26) that could specifically interfere with the binding with AGO2. Our Biacore analyses ascertained binding between the identified peptides and AGO2. Recombinant reporter system Gaussian luciferase and fluorescent bioconjugate techniques were employed to determine biological functions and pharmacokinetic characteristics of these two peptides. Our data suggested that Pep#11 and Pep#26 could function as decoy peptides by mimicking the interaction function of MSI1 with its binding partner AGO2 in vitro and in vivo. Further experiments using GMB animal models corroborated the ability of Pep#11 and Pep#26 in disrupting MSI1/AGO2 interaction and consequently anti-tumorigenicity and prolonged survival rates. These striking therapeutic efficacies orchestrated by the synthetic peptides were attributed to the decoy function to C-terminal MSI1, especially in malignant brain tumors and glioblastoma.

## 1. Introduction

Protein-targeting drugs including various monoclonal antibodies have been proven therapeutically effective in clinics and accumulating evidence also suggests targeting protein–protein interactions that lead to oncogenic developments may be the next era of future medicine for cancers [1,2]. An attractive strategy on targeting protein–protein interaction has based on pinpointing smaller but critical motif(s) within the interacting regions between two binding partners. A series of novel peptides directed against breast cancer stem cell (BCSC) marker GRP78, for instance, was demonstrated to be exceptional in eliminating breast cancer stemness [3]. Antibodies against epidermal growth factor receptors (EGFRs) such as cetuximab and trastuzumab have been used to treat metastatic colorectal cancer and HER2 breast cancer [4,5]. The binding of growth factor EGF to EGFRs is among the most well-studied protein kinase signaling pathways in cancers. Recently, significant breakthroughs have been made into molecular modeling-aided design and discovery of peptide decoys that mimic EGFR ectodomains to block EGF–EGFR interactions [6,7]. Moreover, similar molecular dynamics strategies had also been applied and led to the discovery of Herstatin, a peptide composed of partial HER2 ectodomain that functioned to auto-inhibit HER2-mediated signaling [8]. Another series of peptides designed by molecular dynamics modeling that targets HER2 was shown to be at sub-micro molarities of dissociation constant (*K_D_*) that served as a specific probe by molecular imaging for HER2-positive tumors [9]. To date, such strategies of identifying crucial motifs to disrupt homo- or hetero-dimerization of EGFRs are increasingly proven to be of great potential for clinical use. 

**Figure 1:**
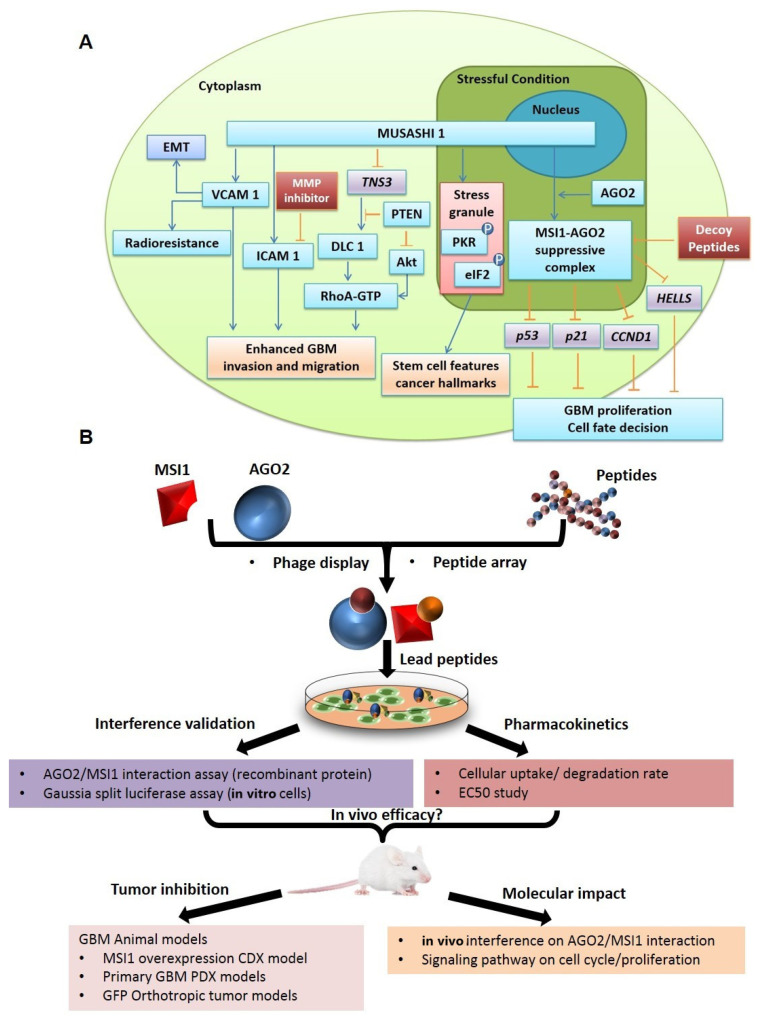
Functional significance of MSI1 in GBM cancer stemness and schematic workflow that illustrates the design, methodology, and key findings from the current study. (**A**) As a member of RNA-binding protein, MSI1 has been functioning to mediate cell fate decision, differentiation, maintenance of stemness for progenitor neural stem cells, and tumorigenesis for cancer cells. The roles of MSI1 have recently been explored regarding involvement in cellular EMT, radioresistance, invasion, and migration as well as downloading signaling pathways of PTEN/Akt, Notch/m-Numb, and PKR/eIF2, etc. The oncogenic formation of MSI1/AGO2 protein complex has also been implicated in promoting GBM tumor progressions. (**B**) The workflow that had been conducted for discovery of unknown peptides that could specifically interfere with MSI1 interaction with AGO2. Key experimental designs utilized for validations of identified peptides are also outlined.

As a member of RNA-binding protein that is abundant in the central nervous system, MSI1 has been shown to function as a predominantly functional marker by governing cell fate decision, differentiation, maintenance of stemness for progenitor neural stem cells, and tumorigenesis for cancer cells [10,11] (Figure 1A). MSI1 has been highlighted for its importance in mediating cellular EMT (epithelial–mesenchymal transition), radioresistance, invasion, and migration via molecules such as VCAM, ICAM, TNS3, and downloading signaling pathway of PTEN and Akt [12,13,14]. The ability of MSI1 to bind with mRNA and translation initiation factors allows translational regulation of its target molecules, thereby suppressing Notch/m-Numb or promoting PKR/eIF2 signaling to control self-renewal capability of cells [15,16,17]. MSI1 was also shown to promote tumor progression as the silencing of MSI1 hampered cancer cell proliferation and apoptosis inhibition [18]. More recently, accumulating reports correlated MSI1 to stemness maintenance in breast and glioma cancer stem cells by downregulating proteasome expression, or by enhancing tumor invasion and migration to regulate cancer radioresistance [12,14,19]. The increasing importance of MSI1 as a prognostic marker in GBM is also reflected by close correlation between MSI1 expression and overall survival rate from high-grade glioma patients [20]. Nonetheless, there is currently no study that strategizes MSI1 as a therapeutic target against any cancer types. A novel regimen that can specifically target aMSI1-associated cancer stem cells (CSCs) populations could lead to effective treatments to refractory tumors.

AGO2 is a membrane-associated cytoplasmic protein known for the catalytic center within the RNA-induced silencing complex (RISC) [21]. AGO2 belongs to another RNA-binding protein (RBP) family, the argonaute subfamily, that is responsible for their pivotal role in RNA silencing processes by mediating mRNA translation and stability of their targets [22,23]. This post-transcriptional control on target genes of AGO2 was achieved by remodeling its occupancy on targets’ 3′-UTR and coding sequence (CDS) region in response to cellular stress [22]. Epidermal growth factor receptor (EGFR), for instance, was demonstrated to enhance its oncogenic role in cancer progression by suppressing microRNA (miRNA) maturation of tumor suppressors when associated with phosphorylated AGO2 under hypoxia [24,25]. The tumorigenic properties of AGO2 through inhibition of target mRNA maturation of tumor suppressors were also established by elevated AGO2 expression in various cancers in addition to GBM [23,26,27,28].

We previously reported that MSI1 mediates stress-induced conditions, such as hypoxia and chemotherapeutic treatments, tumor progression, and recurrence of GBM by translocation to the cytosol and interaction with AGO2 [29]. Through binding to AGO2, the protein complex stabilizes the mRNAs of several cell-cycle promoting genes and promotes the mRNA degradation of several tumor suppressor genes such as p53 and p21. Overexpression of the C-terminus of MSI1 disrupts endogenous MSI1/AGO2 interaction and effectively reduces stress-induced tumor progression [29]. In our present study, therefore, we designed an array of peptides that span the AGO2-interacting region of C-terminal MSI1, aiming to identify smaller companions at around 15 amino acids long that could function as decoys to interfere with protein–protein interaction between MSI1 and AGO2, and to disrupt subsequent oncogenic events in cancer cells thereby conferring therapeutic efficacies for patients with malignant brain tumors and glioblastoma (GBM) (Figure 1B).

## 2. Results

### 2.1. Identifying C-Terminal Motifs Responsible for MSI1/AGO2 Interaction

**Figure 2:**
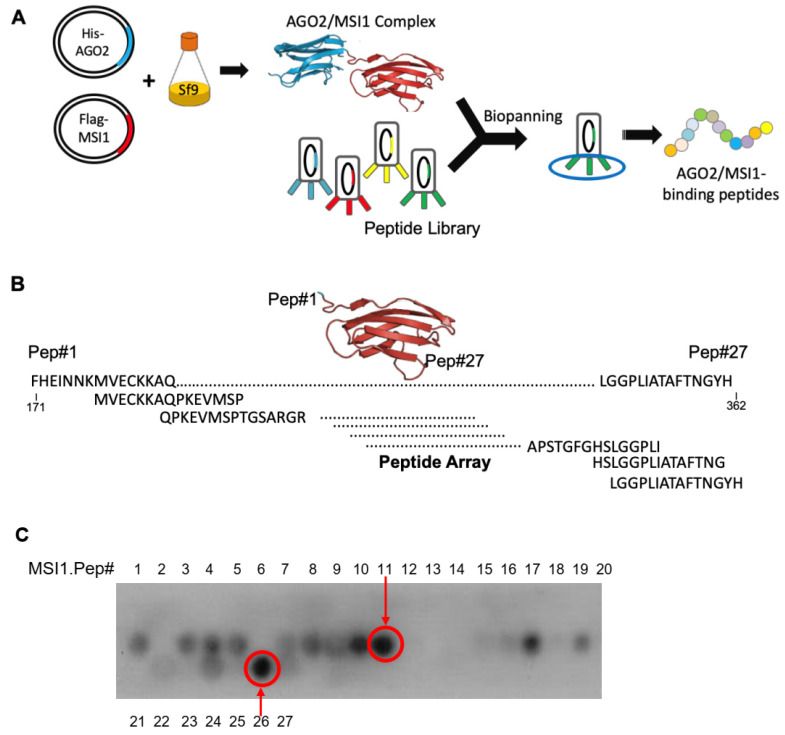
Screening strategy for peptidic motifs from the C-terminus of MSI1 that mimic interaction function with AGO2. (**A**) A schematic cartoon that illustrates a phage display strategy used to identify MSI1 or AGO2 binding peptide. (**B**) A strategy of peptide array that covers the C-terminus of MSI1 (aa 171–362) by 27 sequential peptide sequences of 15 amino acids that overlap with one and another. (**C**) Recombinant AGO2 proteins were incubated with nitrocellulose membrane peptide array dotted with 23 peptide fragments designed from the C-terminus of MSI1. The array revealed two potential interacting peptides with recombinant AGO2.

**Figure 3:**
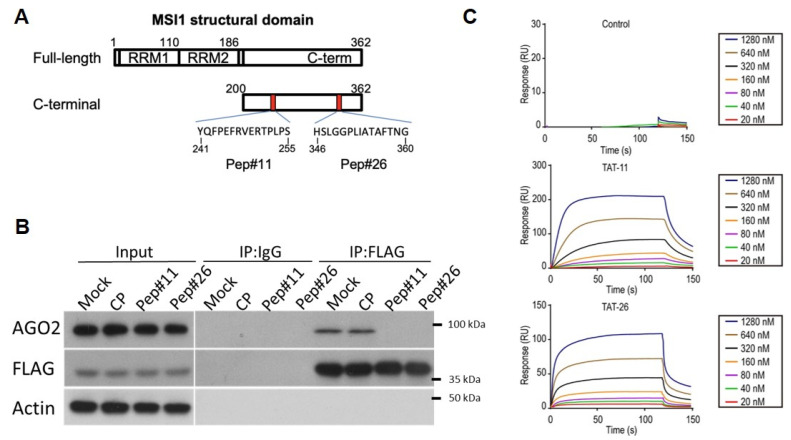
Identification of peptidic motifs from the C-terminus of MSI1 that mimic interaction function with AGO2. (**A**) Structural domain of MSI1 (full-length and truncated C-terminus). (**B**) Cells respectively treated with 10 µM of the two decoy peptides (Pep#11 and Pep#26) or peptide control (CP) were subjected to Co-IP immunoblot to demonstrate the efficacy of the two peptides on blocking the MSI1/AGO2 interaction under hypoxic condition. (**C**) Binding interaction between Pep#11, Pep#26 and their target protein AGO2 was determined by surface plasmon resonance (SPR). The recombinant AGO2 protein was immobilized on CM5 chip and incubated with a serial dilution (from 20 to 1280 nM) of the two peptides as well as a negative control peptide. The association rate constant (ka), dissociation rate constant (kd), and equilibrium dissociation constant (KD) were calculated and presented in Table S2.

Previously, the ability of MSI1 to shuttle into cytosol was revealed, where it functioned to manipulate mRNA stability via its cytosolic binding partner AGO2 thereby promoting cancer progression [29]. Although deletion mutagenesis implicated the potential of the C-terminal fragment of MSI1 (aa 200–362) in tumor suppression, the underlying mechanism remains obscure. In this study, therefore, we first carried out a peptide phage display in an attempt to identify peptides that could interfere with the binding of AGO2/MSI1 (Figure 2A). A total of 19 peptide sequences of 12-mer were identified after biopanning with recombinant AGO2 and MSI1, none appeared to impede the binding between AGO2/MSI1. We next designed a peptide array based on C-terminal MSI1 by securitizing whether any structural motif within this region could be functionally mimicked. The customized peptide array contained 27 sequential peptides that overlap one and another until the entire C-terminus of MSI1 was fully covered (Figure 2B, Table S1). The 27 peptides were then incubated with His-tagged recombinant AGO2 and the immunoblotting results revealed that peptides 11 and 26 from C-terminal MSI1 appeared to be two of the strongest binding peptides to AGO2 among the array (Figure 2C). To investigate whether the identified peptides could indeed bind with AGO2 in cells, we utilized a 13 amino acid cell-penetrating peptide (CPP) from HIV-1 TAT(48–60) and conjugated to our candidate peptides, namely Pep11 and Pep#26, to facilitate their cellular uptake (Figure 3A) [30]. As shown in our co-immunoprecipitation competition experiments, control peptide (CP) could not interfere with the binding complex formation between MSI1 and AGO2 (Figure 3B and Figure S1). In contrast, the MSI1/AGO2 interaction was completely abolished under the presence of Pep#11 or Pep#26. Furthermore, the binding interaction of CP, Pep#11, and Pep#26 with AGO2 was next assessed by surface plasmon resonance (SPR). Two representative Biacore sensorgrams of the binding of the decoy peptides to AGO2 demonstrated that increasing binding equilibrium between the peptides and the recombinant His-tagged AGO2 coupled on sensor chip was monitored as concentration of Pep#11 or Pep#26 was increased from 20 to 1280 nM (Figure 3C). These SPR data determined the equilibrium dissociation constant (*K_D_*) to be 0.515 and 0.674 μM for Pep#11 and Pep#26, respectively. A negative control peptide (CP) was chosen from the weak binding region of MSI1 and revealed a limited affinity (106.6 μM) to AGO2 (Table S2).

### 2.2. Stress-Induced MSI1/AGO2 Binding Complex by Decoy Peptides

**Figure 4:**
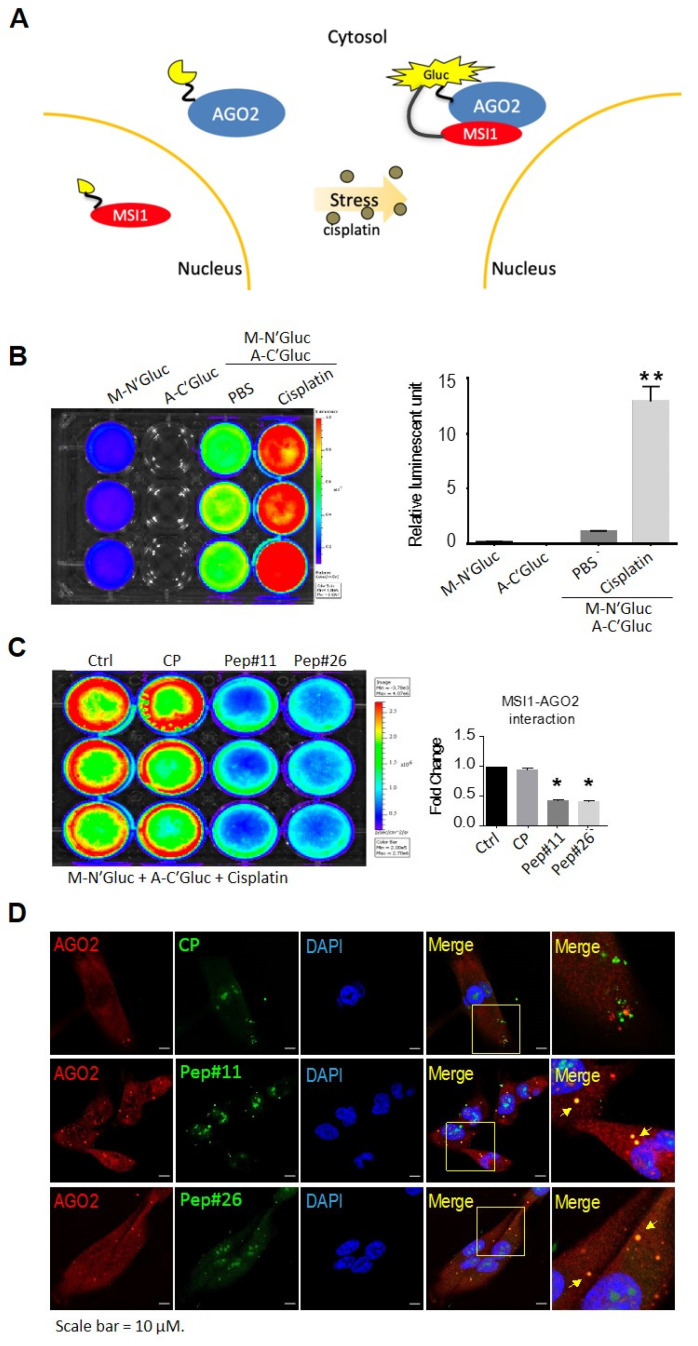
MSI1 C-terminal peptides Pep#11 and Pep#26 acted as decoys for MSI1/AGO2 interaction under cellular stress. (**A**) A schematic illustration that shows in vitro Gaussia luciferase reconstitution assay for the detection of MSI1 and AGO2 protein–protein interaction. (**B**) Gluc-mediated MSI1/AGO2 interaction was quantitatively determined using the split luciferase reconstitution assay as monitored by IVIS imaging system. In total, 30 µM of cisplatin was used to induce cellular stress that led to MSI1/AGO2 interaction. M-N’Gluc (MSI1-N-terminal-Gluc); A-C’Gluc (AGO2-C-terminal-Gluc). **, *p* < 0.01. (**C**) Under the same cellular stress conditions induced by cisplatin, tat-conjugated CP (control peptide), Pep#11, and Pep#26 were employed, and luciferase signals were detected and quantitated by comparison to no treatment control (Ctrl) as normalized results were displayed as a bar chart. *, *p* < 0.05. (**D**) Cells treated with 10 µM of 5′FAM-Pep#11, 5′FAM-Pep#26, or control peptide (5′FAM-CP) were analyzed under confocal microscopy for subcellular co-localizations (MSI1 peptides, green; AGO2, red). Scale bar = 10 μM. In (**B**,**C**), The color spectrum bar represents the intensity of the luciferase activity (luciferase units): the red color indicates strong luciferase activity, meaning a strong protein-protein interaction of MSI1 and AGO2; whereas the blue color indicates weak luciferase activity, meaning a weak interaction between MSI1 and AGO2.

To corroborate whether the identified peptides could act as a decoy, we next developed a split luciferase complementation assay to detect in vivo protein–protein interactions between MSI1 and AGO2. As shown in Figure 4A, Gaussia luciferase (Gluc) was split into two fragments, N-terminus and C-terminus, which were molecularly fused to MSI1 (M-NGluc) and AGO2 (A-CGluc), respectively. Upon MSI1/AGO2 interaction under stress, the two Gluc fragments re-associate to reconstitute its luciferase activity that subsequently leads to light emission in the presence of luciferase substrate. Using an In Vivo Imaging System (IVIS), our data demonstrated that neither M-NGluc nor A-CGluc was able to exert any luciferase luminescence, while cisplatin-treated cells that had been overexpressed with both M-NGluc and A-CGluc showed significantly greater luminescence as compared to PBS-treated group that showed minimal level of luminescence (Figure 4B). Taking advantage of this MSI1/AGO2 interaction assay using IVIS, we next evaluated whether Pep#11 or Pep#26 could act as decoy by interfering with the MSI1/AGO2 interaction. Under cisplatin-induced conditions, the reconstituted luciferase luminescence exerted from Pep#11- or Pep#26-treated cells was significantly lower than those from non-treated or CP-treated cells (Figure 4C). Further, to visualize whether the decoy peptides could interact with AGO2, immunofluorescent staining using 5′FAM-tagged Pep#11 and Pep#26 was conducted. The results confirmed that 5′FAM-Pep#11 and 5′FAM-Pep#26 were both not only capable of entering the cells but were also indeed co-localized with endogenous AGO2 in the cytosol (Figure 4D and Figure S2).

### 2.3. Uptake and Degradation of Decoy Peptides Pep#11 and Pep#26

**Figure 5:**
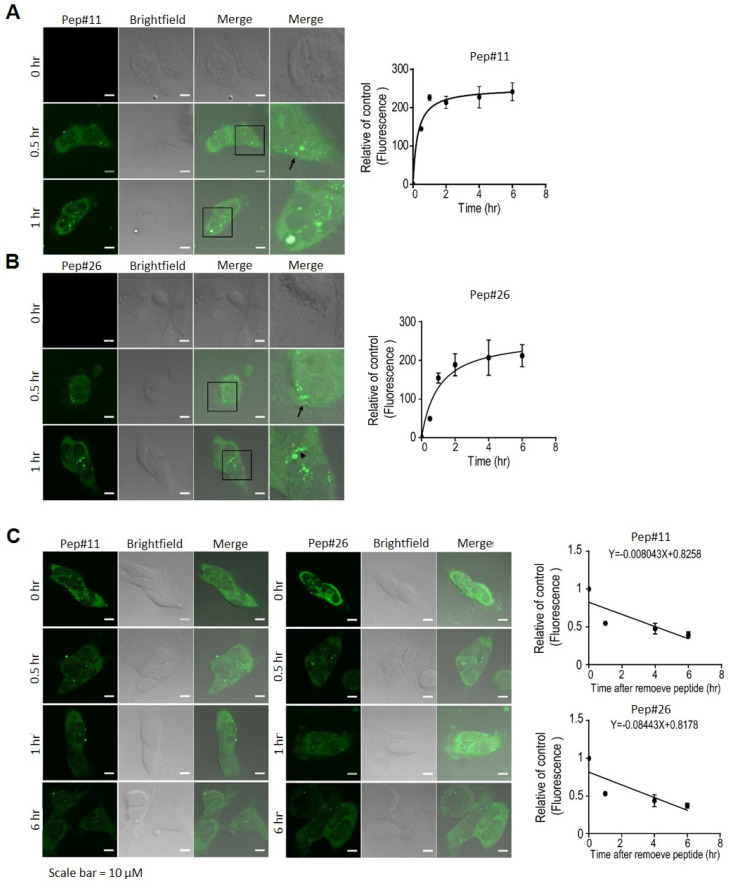
The subcellular localization, cellular intake, and stability of the decoy peptides. (**A**,**B**) DBTRG-05MG cells treated with the 5′FAM-labeled decoy peptides, 5′FAM-Pep#11 or 5′FAM-Pep#26, were analyzed at 0, 0.5, and 1 h by confocal microscopy. Quantitative analyses were performed from fluorescent intensities detected by ELISA reader (*n* = 3 at each time point). (**C**) Cells were respectively treated with two 5′FAM-labeled decoy peptides for up to 6 h. Cells were observed under microscopy and fluorescent intensities were measured by ELISA reader at the indicated time points. To facilitate comparison of intake and degradation dynamics, mean fluorescence values were normalized to stating fluorescence. All data represent three independent experiments. Scale bar = 10 µM.

Prior to investigating therapeutic potentials of the decoy peptides further in vivo, in vitro cellular uptake and degradation rates were measured. The time-lapse confocal microscopy results revealed that significant increases in cellular uptake of Pep#11 and in DBTRG-05MG cells were found within the first hour (Figure 5A); while Pep#26 appeared to show a slightly slower rate of uptake than that of Pep#11 as the highest cellular uptake was reached at 2 h (Figure 5B). The rate at which Pep#11 or Pep#26 was degraded in the cells was also determined by monitoring changes in fluorescence emission from 5′FAM (Figure 5C). The data reasonably showed significant degradation of these 15 amino acid long peptides in the cells within the first half an hour due to cellularly abundant proteases and peptidases, while fluorescence detected after 6 h was likely residual 5′FAM cleaved from peptides.

### 2.4. MSI1 Decoy Peptides Elicit Superior Therapeutic Effects in Tumor Suppression and Prolonging Survival

**Figure 6:**
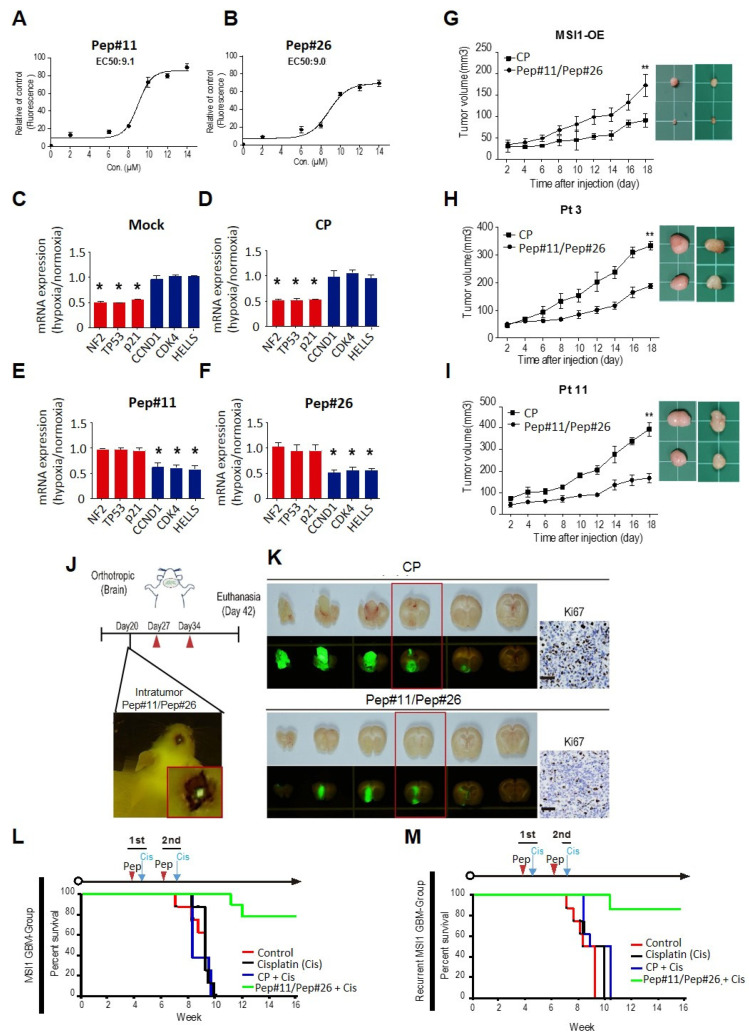
Cellular uptake efficiency and tumor suppression effects of Pep#11/Pep#26. (**A**,**B**) Cellular uptake curves for Pep#11 and Pep#26 peptides. The biological activity of peptides was tested in DBTRG-05MG cell line. The cells were treated with different concentrations of fluorescein labeled peptides and was measured using an ELISA reader. The half-uptake concentration (EC50) values of both peptides were 9.1 and 9.0 μM/mL, respectively. (**C**–**F**) Cells transfected with control peptide (CP), Pep#11, or Pep#26 were under normoxia or hypoxia condition and subjected to qRT-PCR to determine the relative expression level of six downstream targets of the MSI1-AGO2 complex. The mRNA levels under hypoxia versus mRNA levels under normoxia were shown in the bar chart. * *p* < 0.05 in comparison to normoxia. (**G**) DBTRG-05MG/MSI1-wt stable cells were subcutaneously transplanted in immunocompromised mice. Once the tumor mass reached 50 mm^3^, CP or a mixture of Pep#11/Pep#26 (150 µg) was injected at the tumor site six times with 3-day intervals. Tumor size was monitored every 2 days (*n* = 6. ** *p* < 0.01 vs. CP treated control). (**H**,**I**) Immunocompromised mice were subcutaneously transplanted with Pt3 or Pt11 primary GBM cells. Once the tumor mass reached 50 mm^3^, CP or a mixture of Pep#11/Pep#26 (150 µg) was injected at the tumor site six times with 3-day intervals. Tumor size was monitored every 2 days (*n* = 6. ** *p* < 0.01 vs. CP treated control). (**J**) A schematic illustrating the animal experiment design to evaluate the effects of orthotropic delivered Pep#11/Pep#26 (150 µg) on GBM tumor growth. (**K**) Immunocompromised mice were transplanted with GFP-tagged DBTRG-05MG/MSI1-wt stable cells through intracranial injection. Twenty days after transplantation, mice were intracranially injected with control peptide (CP) or Pep#11/Pep#26 (150 µg) for three rounds at 7-day intervals. Mice were sacrificed at day 42 to confirm the GFP tumor signal in the brain. GFP-labeled GBM tumors in serial brain sections of the same mice were observed under fluorescent and optical microscopes. The red boxed tumor sections were subjected to Ki-67 staining and presented in the right panel. Three mice were used in each condition, and the figure showed a representative mouse of each. (**L**,**M**) Survival analyses for mice with orthotopic xenotransplantation of MSI1-overexpressing DBTRG-05MG cells (**L**) or primary cultured tumor cells from recurrent GBM patients (**M**). Mice received two rounds of treatment with a one-week interval of CP or Pep#11/Pep#26 (150 µg) with cisplatin by i.v. injection (*n* = 6).

To determine the efficiency of the decoy peptides on competing AGO2 interaction with endogenous MSI1, FAM was used to label the HIV-TAT-conjugated (TAT-FAM) peptides that were then utilized to assess the uptake rate by measuring concentration of decoy peptide at half-maximal response (EC50) in GBM cells. Our results showed that EC50 concentration for 5′FAM-Pep#11 and 5′FAM-Pep#26 was around 9.1 and 9.0 μM, respectively (Figure 6A,B). We previously reported that under stress condition such as hypoxia, the MSI1/AGO2 complex induced tumor progression by stabilizing the mRNAs of cell cycle promoting genes (*CCND1*, *CDK4*, and *HELLS*) and enhanced the mRNA degradation of cell death related genes (*NF2, TP53*, and *p21*) [29]. This effect was reversed by Pep#11 and Pep#26 transfection in DBTRG-05MG cells (Figure 6C–F), suggesting the peptides not only block MSI1/AGO2 interaction but also block their effects on downstream mRNA targets.

As the EC50 values from Pep#11 and Pep#26 were nearly identical and not in the range of sub-micro molarity, these two decoy peptides were used together to investigate their influence in vivo using MSI1-overexpressed xenografts from DBTRG-05MG cells. As shown in Figure 6G, Pep#11/Pep#26 together demonstrated a significant reduction in tumor growth as compared to xenografts treated with control peptide CP. Next, we further investigated whether the significantly reduced tumor growth could be recapitulated using primary GBM cells (Pt 3 and Pt 11) from patients. Pep#11/Pep#26 again showed pronounced capability in tumor suppression as compared to control CP (Figure 6H,I). Similar results were also observed in the subcutaneous transplanted xenograft model from MIA-PaCa2 pancreatic cancer cells (Figure S3A). 

In addition, an orthotopic xenograft mouse model was developed and the combined Pep#11/Pep#26 peptides were then injected within the tumor sites (Figure 6J). The tumor size in mice treated with Pep#11/Pep#26 peptides was strongly reduced compared to that of the control mice (Figure 6K). Moreover, tumors injected with peptides displayed a severe reduction of Ki67 expression, a marker associated with cell proliferation (Figure 6K, right panel). Similar results on tumor growth were obtained with intraperitoneal injection of Pep#11/Pep#26 peptides in a pancreatic tumor xenograft mouse model (Figure S3B,C). Analysis of the xenograft tumor samples indicated the effectiveness of Pep#11/Pep#26 peptides on tumor growth (Figure S3C), MSI1/AGO2 interaction (Figures S3D and S4), and the downstream mRNA targets (Figure S3E). 

Moreover, based on these observations of tumor suppression capability of Pep#11/Pep#26, we hypothesized these decoy peptides could potentially help with lifespan for xenograft-bearing mice. Interestingly, DBTRG-05MG-bearing mice treated with the peptides along with cisplatin demonstrated significantly higher survival rate as compared to control groups including cisplatin alone and CP plus cisplatin (Figure 6L). Moreover, we further employed the patient-derived xenograft (PDX) established from recurrent GBM tumor. Our results showed that when treated with Pep#11/Pep#26 peptides, the PDX-bearing mice showed significantly greater survival as compared to those without Pep#11/Pep#26 treatments (Figure 6M). These data thus suggested that Pep#11/Pep#26 peptides could enhance the sensitivity of tumor cells to chemotherapy drugs through MSI1/AGO2 interaction blockade.

## 3. Discussion

GBM has been the most notorious brain cancer known for the worst survival of less than two years for patients who even received surgery, chemo- and radiotherapy [31]. Although numerous studies and efforts have been directed towards more therapeutically effective regimens against GBM, there is currently no single therapy or combined regimens that could fully eliminate GBM in clinics [32]. Over the last decade, MSI1 has emerged as an oncogenic protein that functions as a stem cell determinant to promote cancer cell survival, tumor progression, and its expression is closely correlated to drug resistance and cancer relapse in patients [33,34]. Our previous research also suggested subcellular localization of MSI1 could be a diagnostic and prognostic biomarker for GBM patients [29]. Although the C-terminal region of MSI1 was shown to be critical for endogenous MSI1/AGO2 interaction, there is currently no drug that could specifically inhibit the interaction between MSI1 and AGO2. In this study, therefore, we designed peptide arrays in an attempt to discover whether any motifs within the MSI1 C-terminus could act as decoy peptides that are critical for protein–protein interaction with AGO2 (Figure 2A). As a result, Pep#11 and Pep#26 were identified in the arrays and were demonstrated to be effective in not only the MSI1/AGO2 complex disruption in vitro, but in vivo as well where the tumor suppression capability was delineated (Figure 2B,C and Figure 6). The Gluc reporter technology has been widely used for various bioluminescence-based detection applications [35,36]. By utilizing this prominent reporter system constituted from Gaussia luciferase and our two interacting proteins of interest, luminescence signals were restored when recombinant MSI1-Gluc and AGO2-Gluc interacted with each other (Figure 4). This detection system allowed us to substantiate bona fide protein–protein interaction between C-terminal-derived decoy peptides Pep#11/Pep#26 and AGO2 in vitro and in vivo.

Cancers of solid tumors such as GBM are highly malignant, and those patients often suffer from poor prognosis and short survival regardless of regimens [37,38]. In the current study, we demonstrated that the in vivo therapeutic efficacies of the two decoy peptides Pep#11 and Pep#26 were not only effective in cell-line derived GBM xenograft models, but also primary GBM xenografts established from clinical specimens (Figure 6). These pronounced tumor suppression effects exerted by synthetic Pep#11/Pep#26 were likely attributed to the blockade of the oncogenic functions of the endogenous MSI1/AGO2 complex in cancer cells. The region of interference between MSI1 and AGO2 will also be of great interest. The PIWI domain of AGO2 that is known as an evolutionarily conserved catalytic domain conducting endonuclease functions [23] was also shown to allow AGO2 to interact with Dicer to regulate small non-coding RNAs (sncRNAs) processing in mammalian cells [39]. Of note, Pep#11/Pep#26 were the two peptides identified that showed the strongest interaction with recombinant AGO2 protein among at least eight other peptides identified from our peptide arrays. Further work may be required to ascertain whether these peptides could also target the PIWI domain and interfere with the oncogenic functions of AGO2. Critical amino acid residues within these peptides including Pep#11/Pep#26 also remain to be identified for future therapeutics development that bases on improving MSI1/AGO2 interference. Of note, the potential side effects of these peptides on neural progenitor cells are yet to be investigated, though we did not observe any side effects from the brains of treated mice during our experiment.

The sequences of Pep#11 and Pep#26 peptides are specific to MSI1 as they are not conserved in the protein sequence of MSI2. The two peptide sequences are located in the C-terminal domain of MSI1 and are distant by 90 amino acids. However, the specificity of the two peptides and whether there are off target effects of the peptide treatments still need further detailed investigation. Our preliminary prediction of the binding mode between peptides and AGO2 (using molecular docking website (http://galaxy.seoklab.org/index.html, accessed on 13 March 2018) suggested that these two peptides may bind the PIWI domain of AOG2 protein. The PIWI domain is highly conserved in Argonaute protein family, however, only AGO2 is catalytically active and functions as an endonuclease in mammals [23]. Through its PIWI domain, AGO2 also binds to Dicer to regulate the processing of small non-coding RNAs (sncRNA) [39]. The effects of the decoy peptides on the endonuclease activity and sncRNA processing pathways still need to be clarified and further experiments have to be performed to verify whether decoy peptides could interfere with other functions and binding partners of AGO2. Moreover, it is important to determine what amino acids in these peptides are essential for the MSI1/AGO2 interaction for the further sequence optimization and therapeutic development. With our peptide array, we also identified other peptides with a weaker affinity for AGO2; it will be interesting in the future to determine whether these peptides also target the PIWI domain of AGO2 or can recognize other domains in the protein. 

Peptide-based therapeutics that target protein–protein interaction crucial for cancer progression have attracted increasingly enormous attention over conventional small molecules in the past decade [40,41]. Despite a recent study by Cambuli et al. [42] that proposed a v-Msi overexpressing MSI1 specifically in the entire intestinal epithelium for determination of drug metabolism, cell cycle, DNA synthesis, and repair in vivo, no molecules were reported for targeting MSI1. Regarding MSI-targeting, another recent study identified a small molecule Ro 08-2750 that was effective against acute myeloid leukemia (AML) via suppression of highly expressed MSI2 [43]. Therapeutic efficacy of this MSI2 inhibitor on cancers of solid tumor including GBM remains unknown. Nevertheless, numbers of similar peptidic therapeutics strategized from targeting protein–protein interaction in GBM have been reported. For instance, a rising hope from a peptide antagonist that targets αv-integrin receptor through β3 and β5, namely Cilengitide, has entered a phase III trial for treating recurrent GBM. The underlying mechanism by which Cilengitide effectively reduced GBM progression and metastasis was the preferential interruption of EGFRvIII/integrin β3 complex formation [44,45,46]. Interestingly, another recent study of ours that revealed that MSI1 promoted GBM tumorigenesis via upregulation of macrophage inhibitory factor 1 (MIF1) and M2 polarization [47] may provide another implication for the potential application of virus-free gene therapy based on blocking M1/M2 polarization of tumor-associated macrophages (TAMs) [48].

Moreover, although our current study has clearly revealed that the disruption of MSI1/AGO2 interaction is a viable therapeutic approach, Pep#11/Pep#26 did not have an impact on MSI1 shuttling. Thus, further efforts could be directed towards the understanding and development of the prevention of MSI1 cytosolic translocation from the nucleus for GBM patients, whose rate of relapse incidence after chemotherapy desperately needs to be lowered. These examples were clearly in line with our current findings in the synthetic decoy peptides that were demonstrated to convey therapeutic effects on GBM tumors via disruption of the MSI1/AGO2 interaction in vivo. Furthermore, our observations in Pep#11/Pep#26-mediated, dramatically reduced tumor growth of recurrent GMB mice models that had been treated with chemotherapeutic cisplatin (Figure 6) may suggest the potential of conquering drug resistance or relapse after chemotherapy by interfering cisplatin-induced MSI1/AGO2 protein complex formation (Figure 4B).

In summary, peptides Pep#11 and Pep#26 could function by mimicking the MSI1 binding domain to interact with AGO2 in vitro and in vivo. This mimicking ability of Pep#11 and Pep#26 resulted in interference to endogenous MSI1/AGO2 interaction, thereby impairing cellular survival and tumor progression in GMB and PDAC animal models. Our current study thus offered a new rationale for stratifying GBM patients with recurrent tumors.

## 4. Materials and Methods 

### 4.1. Cell Culture 

The human GBM cell line DBTRG-05MG (Denver Brain Tumor Research Group 05), human pancreatic ductal adenocarcinoma cell line (MIA-PaCa2), and their derivative stable cell lines were cultured in Dulbecco’s Modified Eagle’s Medium (DMEM, Life Technologies Inc., Carlsbad, CA, USA) supplemented with 10% fetal bovine serum (HyClone Laboratories Inc., South Logan, UT, USA), 150 g/mL G418 (SIGMA, Cat#A1720), 100 units/mL penicillin, and 100 μg/mL streptomycin (Life Technologies Inc., Carlsbad, CA, USA) under standard culture condition of 37 °C with 95% humidified air and 5% CO2. Cells were sub-cultured with 0.25% trypsin-EDTA (Sigma-Aldrich Co. LLC., St. Louis, MI, USA). All cell lines were tested for mycoplasma contamination. 

The tumor cell cultures were acquired from the Neurological Institute of Taipei Veterans General Hospital. All procedures of tissues acquirements have followed the tenets of the Declaration of Helsinki and are reviewed by Institutional Review Committee at Taipei Veterans General Hospital (2016-09-012C, 2017-07-031B).

### 4.2. Animal Care, Tumor Cell Transplantation, and Non-Invasive Imaging

All procedures involving animals were performed in accordance with the institutional animal welfare guidelines of Taipei Veterans General Hospital (protocol code: 2019-011). For subcutaneous transplantation, cells were harvested in 100 μL PBS and injected subcutaneously into the dorsolateral side of the flank region of 8-week-old male BALB/C nude mice (National Laboratory Animal Center, Taipei, Taiwan) bred and maintained following the Guidelines for Laboratory Animals in the Taipei Veterans General Hospital. Once the tumor mass reached 50 mm^3^, CP or a mixture of Pep#11/Pep#26 (150 µg) was injected at the tumor site six times with 3-day intervals. Tumor size was monitored every 2 days. Six mice were used for each condition in each experiment. For orthotopic transplantation, cells were harvested in 10 μL PBS and injected orthotopic into the brain of 8-week-old male SCID mice (National Laboratory Animal Center, Taipei, Taiwan) bred and maintained according to the Guidelines for Laboratory Animals in the Taipei Veterans General Hospital. After 20 days of transplantation, CP or a mixture of Pep#11/Pep#26 (150 µg) was injected at the tumor site for three rounds with 7-day intervals. Mice were sacrificed at day 42 to confirm the GFP tumor signal in the brain.

### 4.3. PepSpot High-Throughput Peptide Tiling Array and Peptide Phage Display

To rapidly screen the putative binding hotspots along the C-terminus of MSI1, we mimicked the epitope screening method by dotting the synthetic short peptides on nitrocellulose membrane and incubated with purified AGO2 protein [49]. The C-terminus of MSI1 (200–362) was divided into 27 individual synthetic peptides with N-terminal amine attached on the nitrocellulose. Each peptide is 15 amino acids in length and has eight amino acids overlapping with the previous neighboring peptide (Table S1). The purchased PepSpot membrane (JPT peptide technologies, Berlin, Germany) was firstly rinsed in methanol for 5 min, followed by Tris buffer saline supplemented with 0.1% Tween-20 (TBS-T) washing thrice. The membrane was then blocked by Superblock T20 blocking buffer (Thermo Fisher Scientific Inc., Waltham, MA, USA) for 2 h and 2 μg His-tagged AGO2 recombinant protein was added for an overnight incubation. The membrane was washed thrice with TBS-T and incubated with horseradish peroxidase (HRP)-conjugated 6×His tag primary antibody (Genetex Inc., Hsinchu, Taiwan) for 4 h under 4 °C. Enhanced chemiluminescent reagent was used for further blotting.

In phage display experiments, after binding incubation with purified MSI1 and AGO2 recombinant proteins, the selection procedure for phages representing peptides was conducted. Briefly, incubation was carried out for 1 h at 4 °C. The complexes were captured with His-Tag dynabeads (Invitrogen Inc., Carlsbad, CA, USA) and Flag-M1 antibody and washed 10 times at 4 °C. Bound phages were eluted according to manufacturers’ recommendations. Purified phages were then plated on LB agar plates incubated overnight at 37 °C, cultured and precipitated from the culture supernatant with polyethylene glycol (PEG) and re-dissolved in PBS. Plaguing was repeated for four rounds, amplified phage clones were randomly picked and analyzed by ELISA for positive clones followed by DNA extraction and sequencing for peptide sequences.

### 4.4. Split Luciferase Reconstitution Reporter Assay

We applied a gaussia luciferase (Gluc) system to detect protein–protein interaction between MSI1 and AOG2. We split gaussia luciferase into two fragments: the 106 a.a. N-terminus (NGluc) and the 79 a.a. C-terminus (CGluc) [50,51]. After polymerase chain reaction (PCR) amplification, the NGluc and CGluc were subjected to construct fusion protein with MSI1 and AGO2, respectively, in the pcDNA 3.1 and pCMV backbone. Each fusion protein contains a flexible linker (GGGGS) 2 between the protein and polypeptides of split luciferase [52,53]. We stably transfected both fusion protein expression plasmids in DBTRG-05MG GBM cell line and generated a stable cell line with Hygromycin B (Sigma Aldrich Co., St. Louis, MI, USA) and G418 sulfate (Merck Co., Berlin, Germany). We established a normalizing standard by transducing multiple reporter genes into the generated stable cell line for stably expressing green fluorescent protein (GFP), firefly luciferase (FLuc), and herpes simplex virus type I thymidine kinase (HSV1-tk) using lentivirus as previously described [54]. For in vitro MSI1/AGO2 interaction study in cells, cell lysates were prepared with mild reporter lysis buffer (Promega Co., Madison, WI, USA) with a frozen–thaw cycle. The supernatant was then collected after centrifugation and dispensed in a 96-well black flat bottom plate. Coelenterazine (Nanolight Technologies, Ltd., Pinetop, AZ, USA), the substrate of GLuc, was firstly dissolved in methanol and diluted in reporter assay buffer (15 mM potassium phosphate, 25 mM glycylglycine, 15 mM MgSO4, 4 mM EDTA). D-luciferin sodium salt (Promega Co., Madison, WI, USA) was dissolved in sterilized water and diluted in reporter assay buffer supplemented with 2mM ATP. The bioluminescent signals were acquired by Wallac 1420 Victor2 Microplate Reader (Perkin Elmer, Waltham, MA, USA) equipped with auto-dispenser to avoid rapid decay of Gluc. 

### 4.5. Biotinylated Peptide Synthesis and Cell-Penetrating Assay with TAT-Tagged Peptides

In vitro binding assay was carried out with N-terminal biotinylated synthetic peptides (Table S3) based on our peptide array screening. The synthesized peptides (Thermo Fisher Scientific Inc., Waltham, MA, USA) were dissolved in 10% DMSO by 1 mg/mL and subjected to incubation with an equal amount of AGO2 recombinant protein (2 μg each) in T20 blocking solution (Thermo Fisher Scientific Inc., Waltham, MA, USA). After 16 h incubation, the peptides were pulled down with immobilized streptavidin (Pierce 21115, Thermo Fisher Scientific Inc., Waltham, MA, USA). The precipitated peptide/protein complex was subjected to immunoblotting with 6×His primary antibody (GeneTex Inc., Hsinchu, Taiwan) hybridization and detection. Peptide transfection was carried out with Proteojuice (Millipore 71281, Merck Co., Darmstadt, Germany) following the manufacturer’s instruction. For the in vivo compatible cell-penetrating peptide (CPP)-modified peptides, we tested two different CPPs at the C-terminal ends, including TAT (48–60) from HIV [55,56,57].

### 4.6. Binding Affinity Measurements between Decoy Peptides and AGO2

Surface Plasmon Resonance (SPR) was utilized using Biacore T200 (GE Healthcare) to study binding affinities between decoy peptides Pep#11 and Pep#26 peptides with recombinant His-tagged AGO2 protein. The recombinant His-AGO2 and MSI1 peptides were diluted in HBS-P buffer (10 mM HEPES, 150 mM NaCl and 0.005% T20, pH 7.4). To evaluate the binding affinity, recombinant His-AGO2 was immobilized on a CM5 sensor chip (GE Healthcare, BR100012) via amine coupling (~7000 RU) for 3600 s at a rate of 5 µL/mins, and flow rate for binding analysis was run at 30 µL/mins. After injection of each peptide, the surface was regenerated with an injection of 10 mM NaOH. All sensorgrams were double referenced by subtracting the surface effect from the control flow peptide and the buffer effect form the blank buffer. The kinetic values *ka*, *kd,* and *K_D_* were obtained using Biacore T200 Evaluation Software (GE healthcare) assuming the Langmuir 1:1 binding model.

### 4.7. Other General Cellular Molecular Biology Methods

Other general procedures not detailed above including plasmid constructions [29,58], transfection, co-immunoprecipitation, RNA extraction, quantitative real-time PCR (qRT-PCR), gene expression analysis [59,60], Western blotting, immunofluorescence (IF) staining, and immunohistochemistry staining (IHC) [61] were described previously. All antibodies used in this study are listed in Table S4.

### 4.8. Statistical Analysis

Data in the study are expressed as the mean ± SD from at least three independent experiments. The statistical analysis was performed using Student’s *T*-test. Difference was considered significant when * *p* ≤ 0.05 or ** *p* ≤ 0.01.

## 5. Conclusions

Failures of current chemo- and radiotherapy for GBM often attributed to development of resistance to current regimens, the needs for new drugs and therapies that render less undesired resistance in GBM have been imminent. Our present study undertook a combinatorial approach strategized from both molecular dynamics and cell biology methodologies to discover decoy peptides for specific targeting of the MSI1/AGO2 interaction. The two decoy peptides Pep#11 and Pep#26 derived from the C-terminus of MSI1 clearly showed pronounced interference to the binding interaction between MSI1 and AGO2. This interference was not only supported by SPR that suggested *K_D_* at micromolar level but was also demonstrated to be therapeutically effective in treating both GBM tumors derived from cell lines and clinical specimens. Of note, the observation from the treatments of cisplatin/Pep#11/Pep#26 showing significantly greater tumor suppressive effects than cisplatin alone implicated a potential of conquering chemoresistance when MSI1/AGO2 interaction was disrupted. These remarkable anti-tumorigenesis potentials of Pep#11/Pep#26 we identified could be attributed to disruption of the oncogenic functions of MSI1/AGO2.

## Data Availability

The data presented in this study are available on request from the corresponding author.

## Supplementary Materials

The following are available online at https://www.mdpi.com/article/10.3390/cancers14030505/s1, Figure S1: Quantification and original images of Figure 3B, Figure S2: Quantification of the co-localized AGO2 and peptide in Figure 4D, Figure S3: Decoy peptides block MSI1/AGO2 interaction and suppress tumor progression in PDAC animal model, Figure S4: Original images of Figure S3D; Table S1: PepSpot high-throughput peptide array lists, Table S2: Biacore analysis for peptide binding with AGO2, Table S3: N-terminal biotinylated synthetic peptides lists, Table S4: Antibody list.
