# Supplementary material for: Strategic Decoy Peptides Interfere with MSI1/AGO2 Interaction to Elicit Tumor Suppression Effects
Source: Cancers (Basel). 2022 Jan 20;14(3):505. doi: 10.3390/cancers14030505 (PMC8833744; doi:10.3390/cancers14030505)
Supplement: Supplementary file 1 [file cancers-14-00505-s001.zip › cancers-1489371-supplementary.pdf]

Supplementary Materials: Strategic Decoy Peptides Interfere MSI1/AGO2 Interaction to Elicit Tumor Suppression Effects

Yi-Ping Yang, Andy Chi-Lung Lee, Liang-Ting Lin, Yi-Wei Chen, Pin-I Huang, Hsin-I Ma, Yi-Chen Chen, Wen-Liang Lo, Yuan-Tzu Lan, Wen-Liang Fang, Chien-Ying Wang, Yung-Yang Liu, Po-Kuei Hsu, Wen-Chang Lin, Chung-Pin Li, Ming-Teh Chen, Chian-Shiu Chien and Mong-Lien Wang

A

Input				
Ratio (normalized to Actin)	Mock	CP	Pep#11	Pep#26
AGO2	0.932	1.018	1.082	0.886
FLAG	0.928	0.788	0.873	0.794

IP: IgG				
Ratio (normalized to FLAG)	Mock	CP	Pep#11	Pep#26
AGO2	Undetectable			
FLAG	Undetectable			

IP: FLAG				
Ratio (normalized to FLAG)	Mock	CP	Pep#11	Pep#26
AGO2	2.612	2.605	0.000	0.000

B

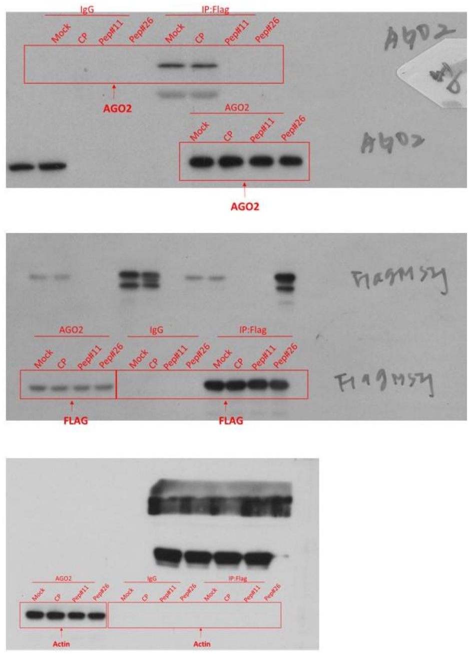

Figure S1. Quantification and original images of Figure 3B. (**A**) The blot intensity of each bends was counted by ImageJ software and normalized. The relative levels are presented in the table. (**B**) The uncropped images of the Western Blot in Figure 3B.

Each cell	AGO2	Merge	Merge/AGO2	STD
CP	8	0	0	0
	4	0	0	
	1	0	0	
Pep#11	16	12	0.75	0.165115
	14	11	0.785714	
	10	8	0.8	
	6	4	0.666667	
	5	2	0.4	
Pep#26	11	6	0.545455	0.09581
	5	3	0.6	
	8	6	0.75	
	7	5	0.714286	

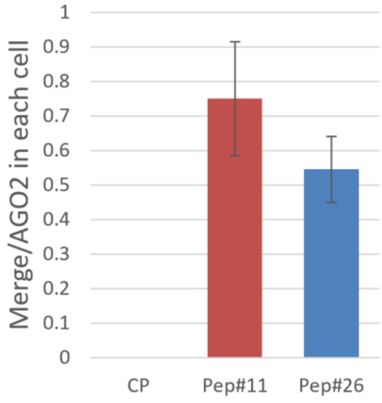

Figure S2. Quantification of the co-localized AGO2 and peptide in Figure 4D. The portion of AGO2 that co-localized with the peptides were calculated. The number of merged yellow puncta, representing signal of the co-localized peptides and AGO2 protein, in Figure 4D was divided by the number of red puncta, representing signal of total AOG2 protein, in each cells (left). The data was shown in the chart (right).

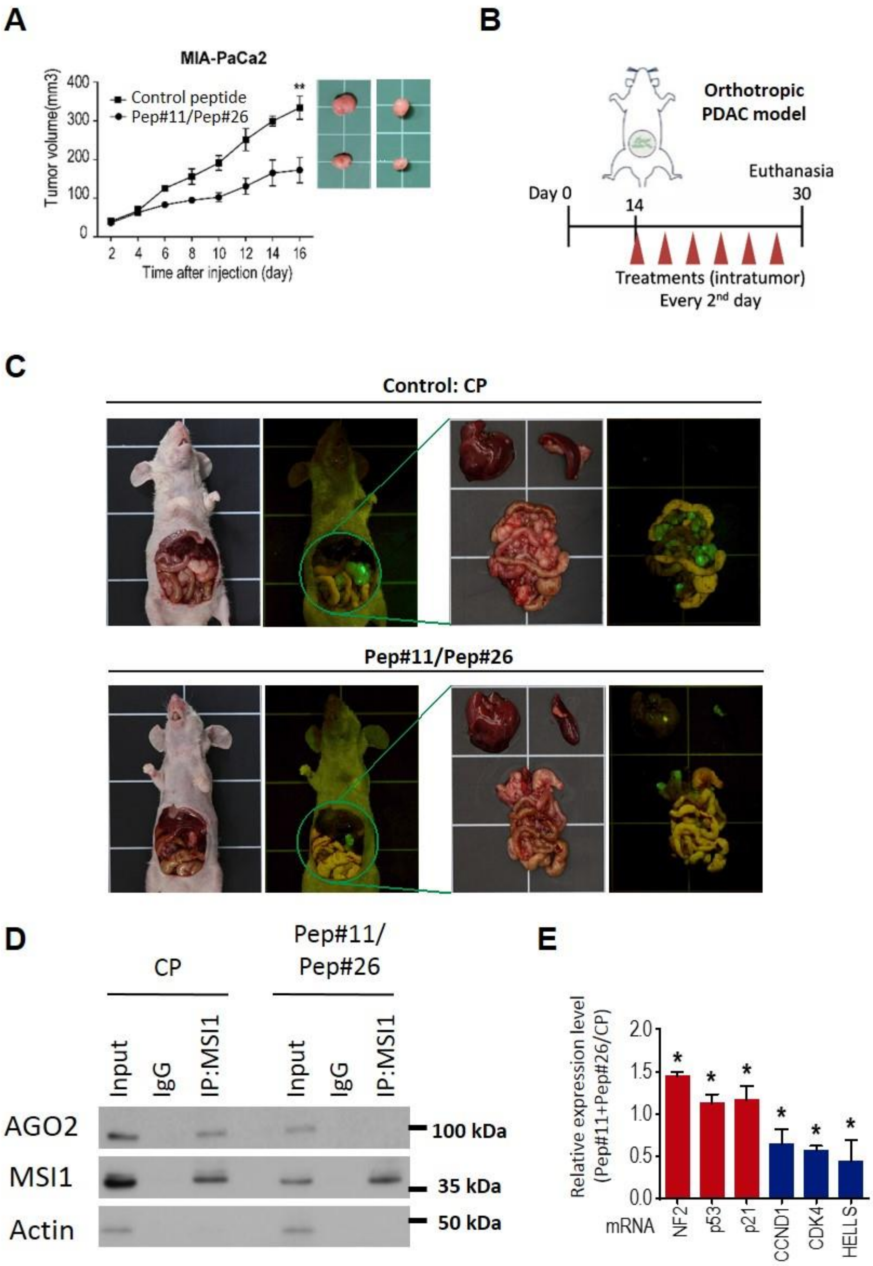

Figure S3. Decoy peptides block MSI1/AGO2 interaction and suppress tumor progression in pancreatic cancer animal model. (**A**) Immunocompromised mice were subcutaneously transplanted with MIA-PaCa2 pancreatic cancer cells. Once the tumor mass reached 50 mm^3, control peptide (CP) or a mixture of Pep#11/Pep#26 (150 μg) was injected at the tumor site 6 times with 3-day intervals. Tumor size was monitored every 2 days. N = 6. ***p* < 0.01 vs. CP group. (**B**) A Schematic illustrating the animal experiment design to evaluate the effects orthotropic delivered Pep#11/Pep#26 (150 μg) on pancreatic tumor growth. (**C**) Immunocompromised mice were transplanted with GFP-tagged MIA-PaCa2 pancreatic cancer cells through intraperitoneal injection. Fourteen days after transplantation, mice were intraperitoneally injected with control peptide (CP) or Pep#11/Pep#26 (150 μg) for 6 rounds with 2-day intervals. Mice were sacrificed at day 30 to confirm the GFP tumor signal. (**D**) The GFP-tagged xenograft tumors were excised and subjected to co-immunoprecipitation assay with anti-MSI1 antibody. (**E**) The GFP-tagged xenograft tumors were analyzed by qPCR to quantify the expression level of downstream target mRNAs. The bar chart shows relative mRNA level in Pep#11/Pep#26-injected mice versus CP-injected mice. **p* < 0.05

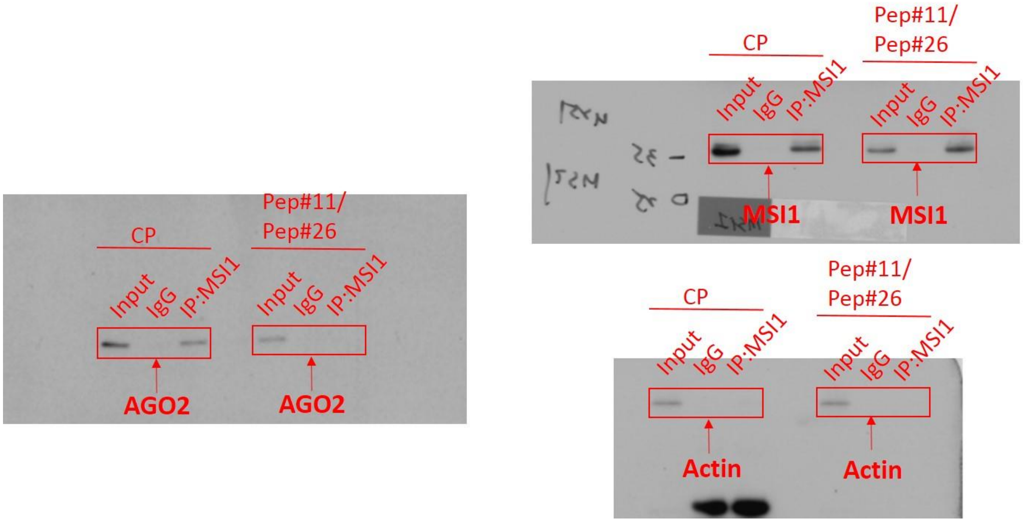

Figure S4. Original images of Figure S3D.

Table S1. PepSpot high-throughput peptide array lists.

Index	Peptide Sequence (N'-C')	Residue No.
1	FHEINNKMVECKKAQ	171–185
2	MVECKKAQPKEVMSP	178–192
3	QPKEVMSPTGSARGR	185–199
4	PTGSARGRSRVMPYG	192–206
5	RSRVMPYGMDAFMLG	199–213
6	GMDAFMLGIGMLGYP	206–220
7	GIGMLGYPGFQATTY	213–227
8	PGFQATTYASRSYTG	220–234
9	YASRSYTGLAPGYTY	227–241
10	GLAPGYTYQFPEFRV	234–248
11	YQFPEFRVERTPLPS	241–255
12	VERTPLPSAPVLPEL	248–262
13	SAPVLPELTAIPLTA	255–269
14	LTAIPLTAYGPMAAA	262–276
15	AYGPMAAAAAAAAVV	269–283
16	AAAAAAVVRGTGSHP	282–290
17	VRGTGSHPWTMAPPP	283–297
18	PWTMAPPPGSTPSRT	290–304
19	PGSTPSRTGGFLGTT	297–311
20	TGGFLGTTSPGPMAE	304–318
21	TSPGPMAELYGAANQ	311–325
22	ELYGAANQDSGVSSY	318–332
23	QDSGVSSYISAASPA	325–339
24	YISAASPAPSTGFGH	332–346
25	APSTGFGHSLGGPLI	339–353
26	HSLGGPLIATAFTNG	346–360
27	LGGPLIATAFTNGYH	348–362

Table S2. Biacore analysis for peptide binding with AGO2.

	k_a (1/Ms)	k_d (1/s)	K_D (μM)
CP	42500	4.529	106.6
Pep#11	32600	0.01684	0.515
Pep#26	3161	0.00213	0.674

Table S3. N-terminal biotinylated synthetic peptides lists.

Tagged Peptides	Sequence (N'-C')
TAT-tagged CP	GSHPWTMAPPPGSTPGRKKRRQRRRPPQ
TAT-tagged Pep#11	YQFPEFRVERTPLPSGRKKRRQRRRPPQ
TAT-tagged Pep#26	HSLGGPLIATAFTNGGRKKRRQRRRPPQ
5′FAM-CP	5′FAM-GSHPWTMAPPPGSTPGRKKRRQRRRPPQ
5′FAM-Pep#11	5′FAM-YQFPEFRVERTPLPSGRKKRRQRRRPPQ
5′FAM-Pep#26	5′FAM-HSLGGPLIATAFTNGGRKKRRQRRRPPQ

Table S4. Antibody list.

ANTIBODIES	SOURCE	IDENTIFIER
Rabbit monoclonal anti-Argonaute 2	Cell Signaling Technology	Cat#2897;
Mouse monoclonal anti-Argonaute 2	Abcam	Cat#ab57113
Rabbit polyclonal anti-Argonaute 2	Abcam	Cat#ab32381
Mouse monoclonal anti-β-Actin	SIGMA	Cat#a5316
Rabbit monoclonal anti-Musashi-1	Cell Signaling Technology	Cat#5663
Rabbit monoclonal anti-Musashi-1	Abcam	Cat#ab52865
Mouse monoclonal anti-Flag M2	SIGMA	Cat#F1804
Mouse polyclonal IgG	Millipore	Cat#12-371
Rabbit polyclonal IgG	Millipore	Cat#12-370
EasyBlot anti-mouse IgG	GeneTex	Cat#GTX225857-01
EasyBlot anti-rabbit IgG	GeneTex	Cat#GTX225856-01
Anti-mouse IgG, HRP-linked Antibody	Cell Signaling Technology	Cat#7076
Anti-rabbit IgG, HRP-linked Antibody	Cell Signaling Technology	Cat#7074
Goat anti-Mouse IgG (H + L) Secondary Antibody, Alexa Fluor 488 conjugate	Invitrogen	Cat#A-11001
Goat anti-Mouse IgG (H + L) Secondary Antibody, Alexa Fluor 555 conjugate	Invitrogen	Cat#A-21424
Goat anti-Rabbit IgG (H + L) Secondary Antibody, Alexa Fluor 488 conjugate	Invitrogen	Cat#A-11008
Goat anti-Rabbit IgG (H + L) Secondary Antibody, Alexa Fluor 568 conjugate	Invitrogen	Cat#A-11036
DAPI	SIGMA	Cat#D9542
